# The Dual Role of Ferroptosis in Cancer: Molecular Mechanisms, Microenvironment Crosstalk, and Precision Therapeutics

**DOI:** 10.3390/cancers18111832

**Published:** 2026-06-03

**Authors:** Yu Zhu, Meijia Chen, Jianglong Chen, Junjie Wang, Rujie Zhou, Yunfei Cui, Guang Li

**Affiliations:** 1 Institute of Medicinal Plant Development, Chinese Academy of Medical Sciences & Peking Union Medical College, Beijing 100193, China; s2023009012@student.pumc.edu.cn (Y.Z.);; 2 Yunnan Branch of Institute of Medicinal Plant Development, Chinese Academy of Medical Sciences & Peking Union Medical College, Xishuangbanna 666100, China; 18043919006@163.com (M.C.);

**Keywords:** ferroptosis, tumor microenvironment (TME), precision oncology, lipid peroxidation, targeted therapy, immune evasion

## Abstract

Ferroptosis, an iron-dependent and lipid peroxidation-driven form of regulated cell death, has emerged as a “versatile player” in oncology. It exerts a dual, context-dependent role in cancer, acting as both a potent tumor suppressor and a facilitator of tumor progression and therapeutic resistance. This review systematically delineates the core molecular regulatory networks of ferroptosis, highlighting the intricate balance between its execution mechanisms—driven by polyunsaturated fatty acid (PUFA) oxidation, iron catalysis, and mitochondrial dysfunction—and the robust endogenous defense systems, including the GSH-GPX4, FSP1/DHODH-CoQ10, and GCH1-BH4 axes. We deeply explore the dichotomous nature of ferroptosis in tumorigenesis: while classical tumor suppressors like p53 and CDKN2A harness ferroptosis to halt tumor growth, cancer cells can hijack lipid metabolic reprogramming and specific enzymes (e.g., iPLA2β) to evade cell death and promote distant metastasis. Furthermore, we dissect the multidimensional crosstalk between ferroptosis and the tumor microenvironment (TME), emphasizing its bidirectional immunoregulatory effects. Although CD8+ T cell-derived IFN-γ can sensitize tumor cells to ferroptosis and amplify anti-tumor immunity, aberrant ferroptotic activation can paradoxically foster an immunosuppressive niche. Finally, we summarize the latest translational strategies using small-molecule inducers and synergistic combination therapies, emphasizing that biomarker-guided patient stratification remains the ultimate paradigm for overcoming resistance and realizing precision ferroptosis-targeted cancer therapy.

## Simple Summary

Cancer remains a major global health challenge, and many patients eventually become resistant to standard treatments that rely on traditional cell death pathways. This article explores a recently discovered process called ferroptosis, an iron-dependent form of cell death caused by the accumulation of damaged fats within the outer layers of cells. We explain that ferroptosis acts as a "double-edged sword" in cancer; while it can naturally stop tumors from growing, some cancer cells learn to bypass this process to survive and spread to other parts of the body. The review also examines how this type of cell death communicates with the tumor’s surrounding environment, which can either help or hinder the body’s natural immune defenses. We conclude by summarizing how new medications designed to trigger ferroptosis, especially when combined with existing therapies, offer a promising way to treat resistant cancers. By understanding these complex biological interactions, scientists can develop more precise and effective personalized treatments. This research is valuable to society because it provides a roadmap for creating the next generation of cancer therapies that can overcome drug resistance and improve patient survival.

## 1. Introduction

Cancer remains a formidable global public health challenge, with its biological complexity and therapeutic resistance standing at the forefront of medical research. According to the 2020 global statistics from the World Health Organization (WHO), there were approximately 19.3 million new cancer cases and nearly 10 million cancer-related deaths, making it the second leading cause of death worldwide [1]. Current standard-of-care modalities, including surgery, chemotherapy, radiotherapy, targeted therapy, and immunotherapy, often face substantial challenges., tumor heterogeneity, acquired resistance, and distant metastasis remain the primary causes of treatment failure [2]. Traditional therapies primarily exert their effects by inducing apoptosis; however, the abnormal inactivation of apoptotic pathways is a key mechanism underlying tumor drug resistance [3,4]. Consequently, exploring non-apoptotic forms of programmed cell death (PCD) has emerged as a crucial breakthrough for overcoming tumor resistance and developing novel anticancer strategies [5,6].

In 2012, Dixon et al. first introduced the concept of “ferroptosis,” defining it as an iron-dependent form of programmed cell death driven by lipid peroxidation [7]. Morphologically, ferroptotic cells lack typical apoptotic features, such as chromatin condensation and the formation of apoptotic bodies. Instead, their hallmark alterations are concentrated in the mitochondria, characterized by a shrunken mitochondrial volume, increased membrane density, reduction or vanishing of mitochondrial cristae, and rupture of the outer mitochondrial membrane [8,9]. Biochemically, ferroptosis is driven by an imbalance in intracellular redox homeostasis, leading to the catastrophic accumulation of lipid peroxides and the depletion of glutathione (GSH) [10]. These distinct morphological and molecular features establish clear boundaries between ferroptosis and other forms of cell death, such as necrosis, pyroptosis, and autophagy-dependent cell death, at the mechanistic level [11].

Research over the past decade has demonstrated that ferroptosis acts as a “versatile player” in oncology, exerting dual effects of both tumor suppression and promotion [12]. On the one hand, ferroptosis serves as a vital endogenous tumor-suppressive mechanism, where various tumor suppressor genes inhibit tumor progression by sensitizing cancer cells to ferroptosis. On the other hand, tumor cells can evade ferroptosis through metabolic reprogramming, and may even hijack factors released during ferroptosis to mediate immune evasion or promote distant metastasis [13]. The complex functions of ferroptosis are closely linked to metabolic heterogeneity within the tumor microenvironment (TME). For instance, the incidence of ferroptosis in melanoma cells is significantly lower in the lymphatic microenvironment compared to the bloodstream, thereby facilitating successful metastatic colonization [14]. This review aims to systematically elucidate the core molecular regulatory networks of ferroptosis, demonstrate its dual role in tumorigenesis, and evaluate its translational potential in precision oncology [10].

## 2. Core Molecular Regulatory Mechanisms of Ferroptosis

The molecular blueprint of ferroptosis is synergistically constituted by core execution processes and upstream regulatory networks. Three major interlocking elements—mitochondrial dysfunction, lipid substrate supply, and iron-mediated catalysis—respectively undertake the oxidative amplification of lipid peroxidation, the provision of reaction substrates, and the core catalytic functions. Together, they form the biochemical execution core of the lipid peroxidation chain reaction. Meanwhile, signaling cascades and epigenetic mechanisms act as upstream regulatory systems, precisely controlling the molecular abundance and activity of these core processes, ultimately determining cellular sensitivity to ferroptosis [15]. The organic integration of these four modules completely delineates the molecular framework governing the onset of ferroptosis in tumor cells [16].

### 2.1. Mitochondrial Dysfunction and Reactive Oxygen Species (ROS) Generation

Mitochondria serve as the core amplifiers in the execution of ferroptosis. Their dysfunction is a hallmark event of ferroptosis, concurrently orchestrating the entire process of iron metabolism, reactive oxygen species (ROS) generation, and lipid peroxidation [17]. In ferroptotic cells, mitochondria exhibit specific morphological alterations, including a shrunken volume, increased membrane density, reduction or vanishing of cristae, and outer membrane rupture, which clearly distinguish them from the mitochondrial damage observed in apoptosis and necrosis [18].

The central role of mitochondria in ferroptosis is primarily manifested in three aspects: First, mitochondrial membrane phospholipids (such as cardiolipin) are rich in PUFAs, making them susceptible to lipid peroxidation catalyzed by lipoxygenases (ALOXs) or cytochrome P450 oxidoreductases (CYPs), thus serving as a crucial source of intracellular lipid peroxides. Second, mitochondria are the central hubs of cellular iron metabolism, participating in heme synthesis and iron-sulfur cluster assembly. Iron overload can generate excessive ROS within mitochondria via the Fenton reaction, directly triggering lipid peroxidation. Third, dysfunctions in the mitochondrial tricarboxylic acid (TCA) cycle and the electron transport chain can further amplify ROS production, exacerbating the lipid peroxidation chain reaction and propelling the ferroptotic process [19,20]. Studies have demonstrated that the deletion of cyclic GMP-AMP synthase (cGAS) leads to a massive accumulation of mitochondrial ROS in tumor cells, significantly exacerbating ferroptosis and inhibiting tumor growth, thereby further validating the central role of mitochondria in ferroptosis regulation [21,22].

### 2.2. Lipid Metabolism: The Fuel for Peroxidation

Lipid peroxidation is the core execution step of ferroptosis, and polyunsaturated fatty acids (PUFAs) are the primary substrates for this process [8,23]. Compared to monounsaturated fatty acids (MUFAs), the conjugated electron cloud effect formed by multiple adjacent carbon-carbon double bonds in PUFA molecules makes the carbon-hydrogen bonds on these double bonds highly susceptible to oxidation. Thus, PUFAs serve as the natural substrates for the lipid peroxidation chain reaction in ferroptosis [24,25]. Free PUFAs cannot directly drive ferroptosis; they must be activated via enzymatic reactions and incorporated into cell membrane phospholipids. This process is primarily coordinated by acyl-CoA synthetase long-chain family member 4 (ACSL4) and lysophosphatidylcholine acyltransferase 3 (LPCAT3) [26].

ACSL4 activates long-chain unsaturated fatty acids (e.g., arachidonic acid [AA] and adrenic acid [AdA]) into acyl-CoA esters [27], which is the crucial initiating step in lipid peroxidation. These activated acyl-CoA esters are the core precursors for the synthesis of arachidonic phospholipids, such as phosphatidylethanolamine-AA (PE-AA) [28], and PE-AA acts as the key substrate for lipid peroxidation in ferroptosis [29,30]. Subsequently, LPCAT3 catalyzes the acyltransferase reaction between the acyl-CoA esters and lysophospholipids, transferring the activated unsaturated fatty acids to the sn-2 position of phospholipids. This forms PUFA-rich phospholipid structures on the cell membrane, significantly enhancing the membrane’s sensitivity to oxidative attacks [26]. The expression levels of ACSL4 and LPCAT3 directly determine the abundance of substrates available for intracellular lipid peroxidation, serving as core determinants of ferroptosis sensitivity [31].

### 2.3. Iron Homeostasis: The Catalytic Core of Peroxidation

The catalytic action of iron ions is the core prerequisite for the continuous amplification of the lipid peroxidation chain reaction, and iron homeostasis dysregulation is a hallmark feature of ferroptosis [32]. The initiation and amplification of lipid peroxidation in ferroptosis rely on the initiating role of the lipoxygenase (ALOX) family and the iron-mediated Fenton reaction [33]. ALOXs, a class of non-heme iron-dependent enzymes, can convert arachidonic acid into hydroperoxides, accomplishing the initiating step of lipid peroxidation. The unstable hydroperoxides are then converted into lipid peroxides (LOOH) through the Fenton reaction catalyzed by ferrous iron (Fe^2+^), thereby triggering the chain amplification reaction of lipid peroxidation [20].

Intracellular iron homeostasis is precisely regulated by proteins associated with iron transport, storage, and release, which collectively determine the size of the intracellular labile iron pool (LIP) [33,34,35,36]. Transferrin (TF) binds to transferrin receptor 1 (TFR1) on the cell membrane, mediating the cellular uptake of extracellular iron; meanwhile, divalent metal transporter 1 (DMT1) is responsible for the transmembrane transport and absorption of intracellular ferrous ions [37]. Ferritin-containing multivesicular bodies (MVBs) act as the core regulators of cellular iron storage and release. They can release ferritin via the exosome pathway or degrade excess ferritin to maintain intracellular iron homeostasis [11]. The level of intracellular free iron is directly correlated with ferroptosis sensitivity: iron chelators (such as deferoxamine, DFO) can inhibit ferroptosis by binding to free iron ions, whereas iron overload significantly enhances cellular sensitivity to ferroptosis. Furthermore, lipocalin-2 (LCN2), an iron-metabolizing protein, promotes the progression of renal cell carcinoma in its iron-bound form, while its iron-free form exerts antitumor activity, directly substantiating the link between iron concentration and ferroptosis [38].

### 2.4. Signaling Cascades and Epigenetic Regulation

Beyond the direct involvement of metabolic substrates, ferroptosis sensitivity is also finely tuned by complex intracellular signaling pathways and epigenetic mechanisms [39]. At the signaling level, the energy-sensing kinase AMPK can phosphorylate acetyl-CoA carboxylase (ACC) during nutrient deprivation, inhibiting fatty acid synthesis and thereby antagonizing ferroptosis. Conversely, the inactivation of the Hippo signaling pathway leads to the nuclear translocation of YAP, which transcriptionally activates pro-ferroptotic genes such as *ACSL4* [40].

At the epigenetic level, histone acetylation and DNA methylation modifications dynamically regulate the transcription of ferroptosis-related genes [15]. For instance, histone deacetylase (HDAC) inhibitors can alter chromatin accessibility to upregulate the expression of pro-ferroptotic factors. Additionally, non-coding RNAs (such as miRNAs and lncRNAs) serve as crucial post-transcriptional regulators. By targeting the mRNA stability of SLC7A11 or GPX4, they finely modulate cellular tolerance to oxidative stress across various tumor contexts. Such a multidimensional regulatory network ensures that ferroptosis is not merely a passive metabolic collapse, but rather a precisely programmed cell death process [41].

## 3. The Endogenous Defense Network of Ferroptosis

Ferroptosis is an iron-dependent, non-apoptotic form of programmed cell death driven by lipid peroxidation; it is not a spontaneous event under normal physiological conditions [7]. Despite the presence of polyunsaturated fatty acid (PUFA) substrates, free iron ions, and mitochondrial metabolic activities required for lipid peroxidation, ferroptosis does not occur spontaneously in normal cells. The core reason is that cells have developed a multi-tiered endogenous defense system. This system continuously maintains cellular redox homeostasis and inhibits the initiation of ferroptosis by scavenging lipid peroxides and blocking the peroxidation chain reaction [16]. Only when endogenous defense systems—such as the GSH-GPX4, FSP1/DHODH-CoQ10, GCH1-BH4, and DHCR7/7-DHC axes—become dysfunctional due to pathological factors or exogenous interventions, is the core execution pathway of lipid peroxidation aberrantly activated. This drives the irreversible accumulation of lipid peroxides, ultimately inducing ferroptosis [42,43,44,45]. In malignant tumors, the abnormal hyperactivation of these defense systems constitutes the core mechanism by which cancer cells evade ferroptosis and develop therapeutic resistance. Consequently, these pathways also provide critical targets for ferroptosis-targeted anticancer therapies [2,10].

These four lines of defense, ranging from classical enzymatic reduction reactions to the fundamental remodeling of membrane structures, constitute a comprehensive defense system with gradient coverage and spatial complementarity. The core components, cellular locations, and functional significance of each defense layer are detailed in Table 1.

### 3.1. The Canonical GSH-GPX4 Axis

The GSH-GPX4 axis is the most central and extensively studied intracellular ferroptosis defense system, serving as the first line of defense against ferroptosis [46]. The core function of this system relies on the synergistic action of system xc^−^, GSH synthesis, and glutathione peroxidase 4 (GPX4) [46]. System xc^−^ is a heterodimeric amino acid transporter composed of SLC7A11 and SLC3A2, which mediates the 1:1 exchange of extracellular cystine for intracellular glutamate [37,49,50]. Upon entering the cell, cystine is reduced to cysteine, which is the rate-limiting substrate for the synthesis of GSH, the primary reductive antioxidant in cells.

GPX4, a selenium-dependent peroxidase, is the core effector molecule of the GSH-GPX4 axis. Utilizing GSH as a cofactor, GPX4 reduces toxic intracellular lipid hydroperoxides into non-toxic lipid alcohols, thereby fundamentally blocking the lipid peroxidation chain reaction. Thus, it is the central intracellular enzyme antagonizing ferroptosis [46,47]. Canonical ferroptosis inducers trigger ferroptosis by targeting different components of this system. Erastin inhibits system xc^−^ to block cystine uptake, leading to GSH depletion and the indirect inhibition of GPX4 activity [51]. In contrast, RSL3 covalently binds to the selenocysteine residue at the active site of GPX4, directly and irreversibly inhibiting its activity to robustly induce ferroptosis [52,53,54]. The Stockwell laboratory first identified these two ferroptosis inducers, Erastin and RSL3, through high-throughput screening, laying the foundation for research into ferroptosis mechanisms [3,55,56,57]. Furthermore, the delivery of a single dose of selenium to the brain can drive GPX4 expression, protecting neurons and improving behavior, further validating the central role of GPX4 in ferroptosis regulation [58,59].

### 3.2. FSP1-CoQ10 and DHODH: GSH-Independent CoQ10 Defense Pathways

The FSP1-CoQ10 and DHODH-CoQ10 pathways constitute the second, GSH-independent line of defense against ferroptosis. When GPX4 is dysfunctional, these pathways antagonize ferroptosis by scavenging lipid peroxides through the redox cycle of coenzyme Q10 (CoQ10) [42,60].

CoQ10 (ubiquinone) is a lipid-soluble antioxidant widely distributed in plant and animal cells [61], with high abundance in organs with significant energy demands. Its reduced form, ubiquinol (CoQH_2_), can directly scavenge lipid peroxyl radical intermediates, thereby halting the lipid peroxidation chain reaction [42]. Ferroptosis suppressor protein 1 (FSP1), localized to the plasma membrane and outer mitochondrial membrane, utilizes NADPH to reduce oxidized CoQ10 to ubiquinol. This continuously regenerates antioxidant activity, forming a ferroptosis defense system independent of GPX4 [62]. Dihydroorotate dehydrogenase (DHODH), localized to the inner mitochondrial membrane (IMM), catalyzes the oxidation of dihydroorotate to orotate while simultaneously reducing mitochondrial oxidized CoQ10 to ubiquinol. This specifically antagonizes lipid peroxidation at the IMM, exerting a ferroptosis defense function that operates in parallel with mitochondrial GPX4 and independently of cytosolic GPX4 or FSP1 [43].

### 3.3. The GCH1-BH4 and DHCR7-7DHC Pathways

The GCH1-BH4 and DHCR7-7DHC pathways form the third line of intracellular defense against ferroptosis. They directly block lipid peroxidation through the synthesis of endogenous lipid-soluble antioxidants while simultaneously modulating the lipid composition of the cell membrane, thereby reducing ferroptosis sensitivity [44,45].

GTP cyclohydrolase 1 (GCH1) is the rate-limiting enzyme in tetrahydrobiopterin (BH4) synthesis, catalyzing the conversion of GTP into BH4 precursors [63]. BH4 is a potent reductive antioxidant that directly scavenges lipid peroxyl radicals. Concurrently, it can remodel the cell membrane lipid composition by regulating fatty acid metabolism, reducing the abundance of oxidizable PUFAs, thus providing a dual mechanism to antagonize ferroptosis. 7-Dehydrocholesterol reductase (DHCR7) is a key enzyme in cholesterol synthesis, responsible for catalyzing the reduction of 7-dehydrocholesterol (7-DHC) to cholesterol in the final step of the pathway [63]. Recent studies have demonstrated that its substrate, 7-DHC, is an endogenous ferroptosis inhibitor that effectively protects phospholipids from autoxidation and fragmentation. The accumulation of 7-DHC confers a ferroptosis-resistant phenotype to tumor cells, enhancing their invasive capacity [45].

### 3.4. The MBOAT System: A New Player in MUFA-Mediated Resistance

The MBOAT system is the most recently discovered fourth line of defense against ferroptosis, representing a novel evasion mechanism independent of classical antioxidant systems. Membrane-bound O-acyltransferase domain-containing 1 and 2 (MBOAT1/2) utilize endogenous or exogenous monounsaturated fatty acids (MUFAs) to remodel cell membrane phospholipids. By replacing membrane PUFAs with MUFAs, they reduce the abundance of lipid peroxidation substrates, thereby antagonizing ferroptosis.

Studies have confirmed that even with the complete loss or dysfunction of GPX4 and FSP1, the activation of the MBOAT system can still sustain tumor cell survival [48]. Its function is highly consistent with the ferroptosis-suppressive effects of protective MUFAs, which is likely associated with cell membrane phospholipid remodeling. The discovery of this system unveils a completely new mechanism by which tumor cells evade ferroptosis via membrane phospholipid remodeling, providing a novel target for overcoming resistance to ferroptosis inducers [48,64].

**Figure 1:**
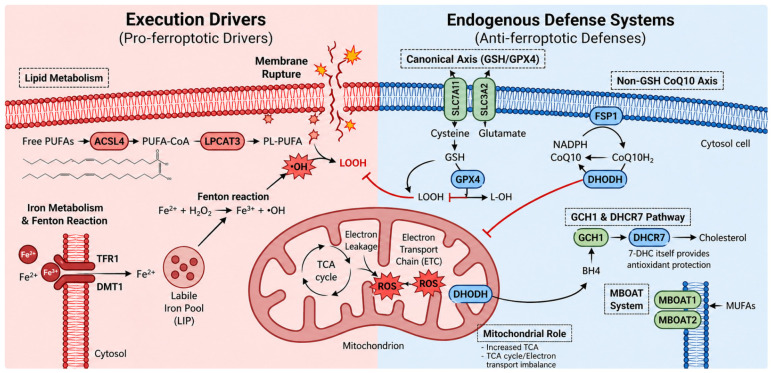
Core molecular execution mechanism and endogenous defense network of ferroptosis. Note: This figure clearly illustrates the three major execution elements of ferroptosis (lipid metabolism, iron homeostasis, and mitochondrial function), the core regulatory pathways of the four major endogenous defense systems, and the crosstalk between these pathways.

In summary, the core molecular execution processes of ferroptosis, together with the four major endogenous defense systems, constitute its comprehensive regulatory network. The synergistic and antagonistic interactions among these pathways directly determine the probability and sensitivity of cellular ferroptosis. The complete mechanism of this regulatory network is illustrated in Figure 1 [15].

## 4. A Double-Edged Sword: The Dual Role of Ferroptosis in Tumorigenesis

Ferroptosis exhibits distinct dual effects in the onset and progression of cancer. On the one hand, it functions as a crucial endogenous tumor-suppressive mechanism, where multiple tumor suppressor signaling pathways can inhibit tumorigenesis by promoting ferroptosis. On the other hand, tumor cells can evade ferroptosis through metabolic reprogramming and may even exploit ferroptosis-related mechanisms to facilitate immune evasion and distant metastasis. This dichotomy is the core reason it is termed a “versatile player” in oncology [12]. This section fundamentally delineates the core pathways underlying ferroptosis as both a tumor-suppressive and a tumor-promoting mechanism. The bidirectional effects of core regulatory factors are summarized in Table 2.

### 4.1. Ferroptosis as a Tumor Suppressor

Ferroptosis is a critical downstream effector mechanism for classical tumor suppressor pathways, such as p53, CDKN2A, and Merlin-Hippo. These tumor suppressor genes exert their antitumor effects by modulating the core ferroptosis pathways, thereby sensitizing tumor cells to ferroptosis [8,15].

p53 is the most frequently mutated tumor suppressor gene in human cancers, and its loss of function is a core driving event in tumorigenesis [72], with high-frequency mutations observed in breast, lung, colorectal, ovarian, and glioblastoma cancers [65,73]. Pioneering work by Jiang et al. demonstrated that p53 transcriptionally represses the expression of SLC7A11 (the core component of system xc^−^), thereby blocking cystine uptake and reducing GSH synthesis, which significantly enhances the sensitivity of tumor cells to ferroptosis [50]. Notably, although the acetylation-defective mutant p533KR loses its ability to induce cell cycle arrest, senescence, and apoptosis, it fully retains its capacity to repress SLC7A11 transcription and induce ferroptosis. This confirms that ferroptosis is a crucial non-apoptotic mechanism through which p53 exerts its tumor-suppressive function [66].

CDKN2A is another vital tumor suppressor gene, frequently deleted in glioblastoma (GBM). Minami et al. discovered that CDKN2A deletion remodels the lipid metabolism of tumor cells, increasing the proportion of PUFAs in cell membrane phospholipids. This significantly elevates lipid peroxidation levels and ferroptosis sensitivity in GBM cells, rendering CDKN2A-deleted GBM cells selectively responsive to ferroptosis inducers [66]. Through alternative splicing, CDKN2A encodes two proteins, p16INK4a and p14ARF, which regulate the CDK4/6-Rb and p53-MDM2 tumor suppressor pathways, respectively, serving as the core defense against uncontrolled cellular proliferation [74]. Although CDKN2A deletion drives cell cycle dysregulation and tumor proliferation, its ferroptosis-sensitizing effect can overwhelmingly counteract the pro-proliferative advantage, making CDKN2A-deleted tumors a key therapeutic target [8].

The Merlin-Hippo-YAP signaling cascade is a highly conserved pathway central to regulating organ size and cell proliferation; its abnormal inactivation is a critical driver of mesothelioma [67]. Merlin (NF2) and LATS2 are core tumor-suppressive components of the Hippo pathway. They phosphorylate YAP/TAZ, sequestering them in the cytoplasm and inhibiting their transcriptional activity. When the Hippo pathway is inactivated, unphosphorylated YAP translocates into the nucleus, binds to TEAD transcription factors, and drives downstream target gene expression [15]. Studies have confirmed that YAP can directly and transcriptionally activate the expression of the key pro-ferroptotic gene *ACSL4* [67], increasing the abundance of lipid peroxidation substrates. This renders mesothelioma cells with inactivated Hippo signaling highly sensitive to ferroptosis. Ferroptosis inducers like sorafenib can selectively eradicate mesothelioma cells by targeting this mechanism and have shown promising efficacy in Phase II clinical trials [75,76].

### 4.2. Ferroptosis in Tumor Promotion and Metastasis

Tumor cells can evade ferroptosis through multiple mechanisms and even hijack ferroptosis-associated pathways to gain a survival advantage, driving tumor progression and distant metastasis. This remains one of the core challenges in ferroptosis-targeted therapies [24].

iPLA2β (calcium-independent phospholipase A2β) is a core molecule that allows tumor cells to evade p53-mediated ferroptosis. iPLA2β preferentially hydrolyzes peroxidized phospholipids on the cell membrane, clearing lipid peroxidation substrates and thereby inhibiting ferroptosis. Although p53 transcriptionally activates the expression of iPLA2β, the two exert diametrically opposed functions in ferroptosis regulation: p53 promotes ferroptosis, whereas iPLA2β acts as a GPX4-independent ferroptosis repressor by clearing peroxidized phospholipids. In vivo studies have demonstrated that inhibiting iPLA2β significantly enhances the sensitivity of melanoma cells to p53-driven ferroptosis and bolsters p53-dependent tumor suppression, suggesting that iPLA2β is a key node for tumor cells to evade ferroptosis [68,77].

Lymphatic circulation is a primary route for distant tumor metastasis, and the lymphatic microenvironment provides a natural “safe haven” against ferroptosis, serving as a core mechanism for the successful metastatic colonization of tumor cells. A milestone study by Ubellacker et al. demonstrated that, compared to tumor cells in the bloodstream, melanoma cells in the lymph exhibit significantly lower oxidative stress and a drastically reduced incidence of ferroptosis, resulting in the formation of more metastatic foci. The underlying mechanism is that lymph contains higher levels of GSH and oleic acid, along with lower concentrations of free iron. Oleic acid can be incorporated into cell membrane phospholipids via an ACSL3-dependent pathway, which reduces the PUFA content, inhibits lipid peroxidation and ferroptosis, and ultimately protects circulating tumor cells during metastasis. This mechanism has also been validated in triple-negative breast cancer (TNBC), where adipocyte-secreted oleic acid inhibits lipid peroxidation and ferroptosis in TNBC cells via the ACSL3-dependent pathway, thereby facilitating their metastasis [69].

### 4.3. Tumor-Type Specificity: Drivers of Ferroptosis Sensitivity and Resistance

While ferroptosis represents a universal cell death modality, the baseline vulnerability to this process varies sharply across different cancer types. This striking tumor-type specificity is fundamentally shaped by distinct intrinsic metabolic dependencies, genetic mutational landscapes, and organ-specific niches.

Highly Sensitive Cancers: Certain tumors exhibit an intrinsic susceptibility to ferroptosis due to their unique lipid metabolism and iron handling profiles. For instance, clear cell renal cell carcinoma (ccRCC) is heavily dependent on lipid accumulation and HIF-2α activation, rendering it exquisitely sensitive to GPX4 inhibition [78]. Similarly, hepatocellular carcinoma (HCC) develops in an iron-rich microenvironment and heavily relies on the SLC7A11-GSH axis to counteract high basal oxidative stress, making it highly responsive to system xc^−^ inhibitors such as sorafenib [79]. Furthermore, therapy-resistant “persister” cells, including targeted therapy-resistant melanoma and breast cancers, frequently acquire a dedifferentiated, mesenchymal-like state. This state is intimately characterized by a dramatically increased abundance of PUFAs in cell membranes, paradoxically creating a robust, targetable vulnerability to ferroptosis inducers.

Highly Resistant Cancers: Conversely, cancers equipped with hyperactive baseline antioxidant defense systems often exhibit profound intrinsic resistance to ferroptosis. A classic example is non-small cell lung cancer (NSCLC) harboring KEAP1 mutations [80]. The loss of KEAP1 leads to the constitutive, hyperactive stabilization of NRF2, a master transcription factor that subsequently upregulates a comprehensive battery of anti-ferroptotic genes, including *SLC7A11*, *FTH1*, and *NQO1* [81]. This confers broad resistance to oxidative stress and most conventional FINs. Additionally, tumors residing in persistently hypoxic niches, or those that endogenously upregulate alternative lipid remodeling pathways (such as the MBOAT1/2 system), can efficiently bypass classical GPX4-dependent defense mechanisms [82].

In summary, mapping the diverse ferroptosis dependencies across the pan-cancer spectrum highlights that future precision oncology strategies must move beyond generic ferroptosis induction. Instead, therapeutic regimens must be rationally tailored to the intrinsic lipidomic and redox profiles of specific tumor types [82].

## 5. Ferroptosis in the Tumor Microenvironment (TME): Crosstalk and Remodeling

The tumor microenvironment (TME) is a complex ecosystem composed of tumor cells, immune cells, stromal cells, extracellular matrix (ECM), and cytokines. Its metabolic heterogeneity and immune landscape directly regulate the ferroptosis sensitivity of tumor cells. Conversely, ferroptosis can actively reshape the immune phenotypes and metabolic profiles of the TME. The multidimensional crosstalk between the two is currently a central hotspot in ferroptosis research and a critical determinant of the efficacy of ferroptosis-targeted therapies [83].

### 5.1. Metabolic Heterogeneity in the TME

The oxygen levels, nutrient composition, and physicochemical properties of the TME exhibit profound spatial heterogeneity, which directly regulates the ferroptosis defense systems and sensitivity of tumor cells.

Oxygen concentration is a crucial microenvironmental factor dictating tumor cell sensitivity to ferroptosis. In hyperoxic environments (such as lung tissue), tumor cells significantly upregulate the expression of the iron-sulfur cluster biosynthetic enzyme NFS1. NFS1 maintains the stability of intracellular iron-sulfur (Fe-S) clusters. A deficiency in Fe-S clusters robustly activates the iron-starvation response, which, when combined with the inhibition of GSH synthesis, triggers ferroptosis. Therefore, tumor cells in hyperoxic environments acquire ferroptosis resistance by upregulating NFS1, making NFS1 a specific therapeutic target for tumors in oxygen-rich microenvironments [70].

The metabolic disparities between the lymphatic and blood microenvironments are the core reasons for the differential ferroptosis sensitivity of circulating tumor cells (CTCs). As previously mentioned, the high abundance of oleic acid and GSH, coupled with low levels of free iron in the lymph, confers ferroptosis protection to cells of melanoma, triple-negative breast cancer (TNBC), and other tumors. This enables them to survive in circulation and successfully complete distant metastasis. In contrast, the higher oxidative stress levels and free iron concentrations in the blood microenvironment make tumor cells more susceptible to ferroptosis. This explains why tumor cells exhibit a stronger propensity to disseminate early via the lymphatic circulation [14].

### 5.2. Ferroptosis and Anti-Tumor Immunity

There is a pronounced synergistic effect between ferroptosis and anti-tumor immunity. The two form a positive feedback loop of “immune activation–ferroptosis enhancement,” which serves as the core theoretical foundation for combining ferroptosis inducers with immunotherapy [84].

CD8+ cytotoxic T cells are the core effector cells of anti-tumor adaptive immunity. They can directly regulate the ferroptosis sensitivity of tumor cells by secreting interferon-gamma (IFN-γ). Pioneering research by Wang et al. demonstrated that IFN-γ secreted by CD8+ T cells significantly downregulates the expression of SLC7A11 and SLC3A2, the two core subunits of system xc^−^ in tumor cells. This blocks cystine uptake, inhibits GSH synthesis, and promotes lipid peroxidation and ferroptosis in tumor cells [85]. This discovery was the first to reveal that adaptive immunity can exert its cytotoxic effects by inducing tumor cell ferroptosis, establishing a direct link between T cell-mediated anti-tumor efficacy and ferroptosis.

Simultaneously, ferroptotic tumor cells can release tumor-associated antigens (TAAs) and damage-associated molecular patterns (DAMPs), functioning as a form of immunogenic cell death (ICD). This activates the antigen-presenting function of dendritic cells (DCs), promotes the tumor infiltration and activation of CD8+ T cells, and further amplifies the anti-tumor immune response [13]. Studies have confirmed that tumor tissues with high ACSL4 expression exhibit a significantly higher abundance of CD8+ T cell infiltration, and these patients show superior response rates to immune checkpoint inhibitors (ICIs) [86]. Conversely, ACSL4 deficiency significantly impairs T cell-mediated anti-tumor immune responses [31], solidifying the synergistic interplay between ferroptosis and anti-tumor immunity.

### 5.3. Pro-Tumorigenic Effects Within the TME

Ferroptosis also exerts pro-tumorigenic effects within the TME. It can promote tumor progression by impairing the function of anti-tumor immune cells and inducing the formation of an immunosuppressive microenvironment, which further highlights its role as a “versatile player.”

Myeloid-derived suppressor cells (MDSCs) and tumor-associated neutrophils are the core immunosuppressive cells within the TME. Kim et al. found that pathologically activated neutrophils (polymorphonuclear MDSCs, PMN-MDSCs) in the TME undergo ferroptosis. Although their numbers decrease, these ferroptotic PMN-MDSCs release large quantities of immunosuppressive molecules, such as oxygenated lipids, which significantly inhibit T cell proliferation and effector functions, paradoxically promoting tumor progression [87]. These results indicate that the cytotoxic effect of ferroptosis on immunosuppressive cells can be completely offset by the resulting T cell suppression, ultimately culminating in a pro-tumorigenic outcome [88].

IL-1β, a core pro-inflammatory cytokine in the TME, exhibits a dual role in ferroptosis regulation. On the one hand, IL-1β recruits neutrophils and monocytes to the tumor tissue, enhancing the cytotoxic functions of T cells. On the other hand, IL-1β can activate the PCAF-NNT axis, augmenting the enzymatic activity of nicotinamide nucleotide transhydrogenase (NNT) and increasing NADPH production. This maintains the stability of Fe-S clusters in tumor cells, protecting them from ferroptosis and immunotherapy-induced cytotoxicity. Clinical data show that higher acetylation levels of NNT at K1042 in the tumor tissues of gastric cancer patients correlate with advanced disease stages and poorer overall survival (OS). This validates that IL-1β-mediated ferroptosis evasion is a crucial mechanism of resistance to tumor immunotherapy [89,90].

Furthermore, aberrant lipid metabolism within the TME can directly induce ferroptosis in CD8+ T cells, impairing their anti-tumor functions. Studies have demonstrated that the expression of the fatty acid transporter CD36 is significantly upregulated on the surface of tumor-infiltrating CD8+ T cells. This mediates an excessive uptake of fatty acids from the TME, inducing lipid peroxidation and ferroptosis, which results in a reduction in cytotoxic cytokine secretion and compromised anti-tumor efficacy. Blocking CD36 or inhibiting ferroptosis can significantly restore the anti-tumor activity of CD8+ T cells, and when combined with anti-PD-1 antibodies, elicits a more potent anti-tumor response [71].

**Figure 2:**
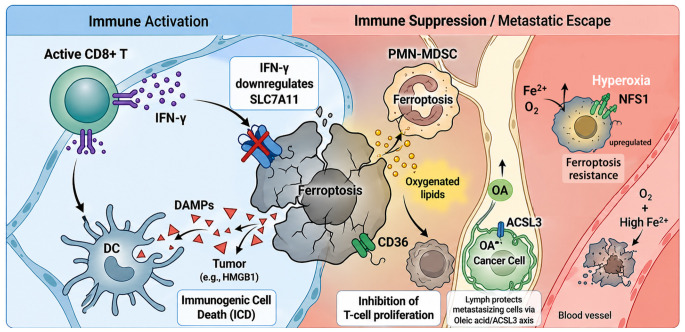
Dual biological effects of ferroptosis in tumorigenesis and development and its multidimensional interaction with the tumor microenvironment. Note: This figure systematically depicts the tumor-suppressive and pro-tumorigenic effects of ferroptosis, alongside its bidirectional crosstalk mechanisms with immune cells and the metabolic microenvironment within the TME.

In summary, ferroptosis exerts bidirectional biological effects in cancer—both tumor-suppressive and tumor-promoting. Its ultimate functional trajectory is inextricably linked to the intrinsic genotype of tumor cells, as well as the metabolic and immune landscapes of the tumor microenvironment. The comprehensive mechanisms of ferroptosis in tumorigenesis, development, and TME crosstalk are illustrated in Figure 2.

## 6. Translating Ferroptosis into Precision Oncology

Based on the core regulatory mechanisms of ferroptosis and its dual effects in cancer, targeting ferroptosis has emerged as a central direction in the development of novel anti-cancer therapies. Currently, various ferroptosis inducers have demonstrated remarkable anti-tumor activity in preclinical models. However, optimizing combination therapeutic strategies and implementing patient stratification based on biomarkers remain the core pathways to successfully realize the clinical translation of ferroptosis-targeted therapies.

### 6.1. Small-Molecule Ferroptosis Inducers (FINs)

Small-molecule ferroptosis inducers (FINs) are the cornerstone drugs for ferroptosis-targeted therapy. They primarily exert their anti-tumor effects by inhibiting endogenous ferroptosis defense systems and augmenting lipid peroxidation. Currently, FINs are mainly classified into two major categories [91]:

System xc^−^ Inhibitors: These are the most classical FINs, triggering ferroptosis by blocking cystine uptake and depleting GSH. Erastin was the first discovered system xc^−^ inhibitor, capable of specifically inhibiting system xc^−^ activity to induce ferroptosis in tumor cells. To improve the druggability of Erastin, researchers developed its derivative, imidazole ketone erastin (IKE). Compared to Erastin, IKE exhibits superior metabolic stability and in vivo anti-tumor activity. Delivering IKE via polyethylene glycol-poly(lactic-co-glycolic acid) (PEG-PLGA) nanoparticles can further reduce its bio-toxicity and enhance tumor targeting. Furthermore, nanoparticles co-loaded with doxorubicin and Erastin can effectively eradicate both cancer stem cells and bulk tumor cells by synergistically modulating redox homeostasis. Sorafenib, an FDA-approved multi-kinase inhibitor, also functions as a potent system xc^−^ inhibitor. It can induce ferroptosis by inhibiting SLC7A11, showing significant efficacy in tumors such as hepatocellular carcinoma (HCC) and mesothelioma. Additionally, sulfasalazine is another approved system xc^−^ inhibitor that can suppress the proliferation and migration of bladder cancer cells by inducing ferroptosis [92].

GPX4-Targeted Inhibitors: These agents directly and irreversibly inhibit GPX4 activity, thoroughly blocking the clearance of lipid peroxides. They are currently the most potent known FINs [93]. RSL3 is the first identified covalent GPX4 inhibitor; it binds to the selenocysteine residue at the active site of GPX4, causing irreversible inactivation and demonstrating robust selective cytotoxicity in RAS-mutant tumor cells. To overcome the pharmacological drawbacks of RSL3 (e.g., poor aqueous solubility and low in vivo stability), researchers have developed novel GPX4 inhibitors, such as ML162 and ML210, which possess higher potency and target selectivity. Currently, the primary challenge in developing GPX4 inhibitors lies in their narrow therapeutic window. Because GPX4 plays essential physiological roles in normal tissues (such as the kidneys and nervous system), systemic GPX4 inhibition can lead to severe toxic side effects. Developing tumor-specific delivery systems (such as antibody-drug conjugates [ADCs] and tumor-targeted nanoparticles) is the core strategy to resolve this hurdle [91,94].

### 6.2. Synergistic Combination Therapies

Given the diverse ferroptosis evasion mechanisms and pronounced tumor heterogeneity, single-agent FIN therapies are prone to acquired resistance [95]. Combination therapeutic strategies can significantly enhance anti-tumor efficacy by synergistically amplifying oxidative stress, blocking evasion pathways, and remodeling the immune microenvironment. This represents the mainstream direction for future clinical applications [95,96].

FINs Combined with Chemotherapy/Radiotherapy: One of the core cytotoxic mechanisms of traditional chemotherapy and radiotherapy is the induction of ROS generation, leading to oxidative stress and DNA damage in tumor cells. FINs can suppress the antioxidant defense systems of tumor cells, shattering their redox homeostasis. This robustly amplifies the oxidative damage induced by chemo/radiotherapy, achieving a synergistic cytotoxic effect while reversing chemo/radio-resistance. Furthermore, this combination allows for a significant reduction in the required dosage of single therapies, thereby mitigating treatment-related toxicities [95].

FINs Combined with Immune Checkpoint Inhibitors (ICIs): This is currently the combination strategy with the greatest potential for clinical translation. As previously discussed, FINs can induce immunogenic ferroptosis in tumor cells, releasing tumor antigens and DAMPs to activate adaptive anti-tumor immunity [96]. Conversely, ICIs (such as anti-PD-1/PD-L1 antibodies) can relieve T cell immunosuppression, further amplifying IFN-γ-mediated tumor ferroptosis. This establishes a robust positive feedback loop of “ferroptosis–immune activation.” Preclinical studies have validated that GPX4 inhibitors combined with anti-PD-1/PD-L1 antibodies exhibit anti-tumor efficacies vastly superior to monotherapies across multiple tumor models, including triple-negative breast cancer (TNBC) and HCC [97,98].

Metabolic Interventions Combined with FINs: Targeting the metabolic evasion pathways of tumor cells can significantly enhance the efficacy of FINs. For instance, CPT1A is the key rate-limiting enzyme in mitochondrial fatty acid oxidation. It can form a positive feedback loop with c-Myc to activate the NRF2/GPX4 system, bolstering ferroptosis resistance in tumor cells. Targeted inhibition of CPT1A dismantles this protective mechanism, resensitizing tumor cells to ferroptosis, and its combination with immunotherapy yields markedly enhanced efficacy [99]. Additionally, targeted inhibition of ACSL3 can block the oleic acid-mediated ferroptosis protection, suppressing lymphatic metastasis and colonization of tumor cells, making it a highly promising combination strategy for metastatic tumors [14].

### 6.3. Biomarkers for Patient Stratification

Precise patient stratification is the fundamental prerequisite for maximizing the efficacy and minimizing the toxicity of ferroptosis-targeted therapies. Identifying biomarkers associated with ferroptosis is the key to screening the populations most likely to benefit. To date, several predictive biomarkers with high clinical translational potential have been discovered [100]:

ACSL4: As the core execution gene of ferroptosis, the expression level of ACSL4 directly determines the abundance of lipid peroxidation substrates within tumor cells. Therefore, it serves as a positive predictive biomarker for ferroptosis sensitivity [37]. Tumors with high ACSL4 expression possess ample lipid peroxidation substrates, making them significantly more sensitive to FINs. Simultaneously, these patients tend to exhibit a superior response to ICIs.

SLC7A11: As the core subunit of system xc^−^, the expression level of SLC7A11 reflects the GSH synthesis capacity and antioxidant defense level of tumor cells, functioning as a negative predictive biomarker for ferroptosis sensitivity [37]. Tumors with high SLC7A11 expression exhibit enhanced cystine uptake and GSH synthesis, rendering them insensitive to GPX4 inhibitors. However, they represent ideal targets for system xc^−^ inhibitors.

Other Biomarkers: Additionally, p53 mutation status, CDKN2A deletion, NFS1 expression levels, and Hippo pathway activity can all serve as predictive biomarkers for tumor ferroptosis sensitivity [8,49]. For example, tumors harboring wild-type p53 retain the ferroptosis regulatory capacity mediated by the p53-SLC7A11 axis, thereby responding better to system xc^−^ inhibitors. Conversely, CDKN2A-deleted glioblastomas exhibit selective sensitivity to FINs. Moving forward, the development of integrated biomarker panels based on multi-omics data is the central direction for realizing personalized applications of ferroptosis-targeted therapies. Such approach would surpass the limitation of relying on a single gene expression to predict complex redox and lipidomic responses.

**Figure 3:**
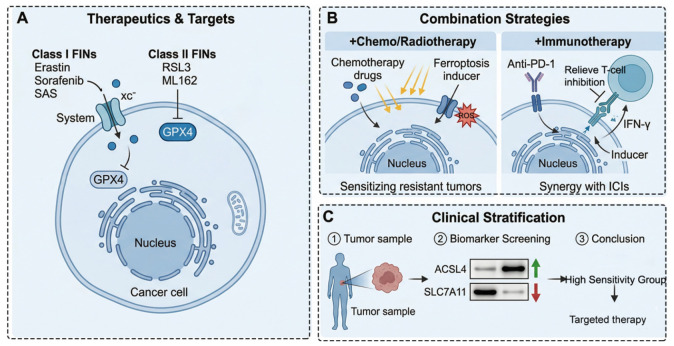
Clinical translation strategy system of ferroptosis-targeted precision cancer therapy. Note: This figure comprehensively illustrates the three core translational directions for targeting ferroptosis: small-molecule drug discovery, synergistic combination therapeutic regimens, and patient stratification biomarker systems, along with the key sub-strategies for each direction. (**A**) Therapeutics & Targets; (**B**) Combination Strategies; (**C**) Clinical Stratification.

In summary, the clinical translation framework for targeting ferroptosis encompasses three core dimensions: small-molecule drug development, combination therapy optimization, and biomarker-guided precise stratification. The complete clinical translation strategy framework is illustrated in Figure 3.

## 7. Current Challenges and Future Perspectives

Although targeting ferroptosis has introduced a novel and promising avenue for cancer therapy, its translation from basic research to clinical practice still faces several core challenges. Despite the formidable pipeline of preclinical ferroptosis inducers, a fundamental and unresolved gap exists between laboratory findings and successful clinical trials. Currently, most active clinical anti-cancer studies incorporating ferroptosis as a parameter still lack rigorous, reliable, and biomarker-driven patient stratification. Identifying which patient subpopulations will genuinely benefit from these therapies, while ensuring acceptable safety profiles, remains the ultimate paradigm that requires urgent clarification. Addressing these translational hurdles will be the central focus of future research in order to realize the full potential of precision ferroptosis oncology.

### 7.1. Overcoming Acquired Resistance

The acquired resistance of tumor cells remains the primary challenge facing ferroptosis-targeted therapies [2]. Tumor cells can develop resistance to ferroptosis through multiple mechanisms: First, the activation of alternative antioxidant defense systems. For instance, upon GPX4 inhibition, tumor cells may upregulate the expression of FSP1 and GCH1 to compensatorily enhance their capacity to scavenge lipid peroxides. Second, lipid metabolic reprogramming. By activating the MBOAT system and downregulating ACSL4 expression, tumor cells reduce the PUFA content in cell membranes, thereby decreasing the abundance of lipid peroxidation substrates. Third, iron homeostasis remodeling. By downregulating TFR1 and upregulating ferritin, tumor cells deplete the intracellular labile iron pool, thereby suppressing the Fenton reaction. Moving forward, profoundly deciphering the molecular mechanisms of acquired ferroptosis resistance and developing novel inhibitors against these resistance nodes represent the core strategies to overcome this obstacle.

### 7.2. Minimizing Off-Target Toxicity

A major bottleneck in ferroptosis-targeted therapy is its narrow therapeutic window and significant off-target toxicity [94]. The core regulatory molecules of ferroptosis (e.g., GPX4, system xc^−^) fulfill essential physiological functions in normal tissues. Systemic administration can trigger aberrant ferroptosis in healthy tissues, leading to severe side effects such as acute renal injury and neurotoxicity. In the future, the development of tumor-specific delivery systems will be the primary strategy to resolve this issue. Advanced platforms, including tumor-targeted nanoparticles, antibody-drug conjugates (ADCs), and TME-responsive prodrugs, can achieve the specific accumulation of ferroptosis inducers (FINs) within tumor tissues. This approach maximizes anti-tumor efficacy while significantly mitigating toxic side effects in normal tissues [101,102].

### 7.3. Deeply Deciphering the Context-Dependent Function of Ferroptosis

The “versatile player” effect of ferroptosis in cancer is highly context-dependent. Tumor cells with varying cancer types, genetic backgrounds, and within different tumor microenvironments exhibit significantly different responses to ferroptosis [103]. For example, FINs demonstrate robust anti-tumor activity in hematological malignancies, hepatocellular carcinoma, and renal cell carcinoma, but show limited efficacy in certain other cancer types. Concurrently, while eradicating tumor cells, ferroptosis may also impair the function of anti-tumor immune cells, mediating immunosuppression [88]. Therefore, future studies must deeply decipher the specific regulatory networks of ferroptosis across diverse oncological contexts. Clarifying the boundary conditions between its tumor-promoting and tumor-suppressive effects is crucial for designing precision therapeutic strategies that maximize tumor suppression while circumventing pro-tumorigenic risks.

## 8. Concluding Remarks

In summary, ferroptosis, as a distinct iron-dependent and non-apoptotic form of programmed cell death, acts as a “versatile player” in the initiation, progression, metastasis, and therapeutic response of cancer, exerting both tumor-suppressive and tumor-promoting effects. Research over the past decade has systematically unraveled the core molecular regulatory networks, endogenous defense systems, and multidimensional crosstalk with the TME, establishing a solid theoretical foundation for ferroptosis-targeted cancer therapies.

Targeting ferroptosis provides entirely new strategies for overcoming apoptosis-resistant tumors, improving immunotherapy responses, and suppressing distant metastasis. Indeed, numerous FINs and combination regimens have exhibited remarkable anti-tumor activities in preclinical models. Nevertheless, the dual biological effects of ferroptosis, alongside tumor heterogeneity, acquired resistance, and off-target toxicity, remain the core challenges impeding its clinical translation. Moving forward, patient stratification based on biomarkers, the development of tumor-specific delivery systems, and the optimization of novel combination therapies will be the pivotal directions for realizing the clinical translation of ferroptosis-targeted treatments. As research deepens, targeting ferroptosis is poised to occupy a prominent position in the future blueprint of precision oncology, bringing renewed hope to cancer patients.

## Figures and Tables

**Table 1:** Hierarchical architecture and core functions of the endogenous ferroptosis defense network.

Defense Layer	Core Axis/Enzyme	Primary Antioxidant	Cellular Location	Functional Significance	References
Layer 1	GSH-GPX4	Glutathione (GSH)	Cytosol/Mitochondria	Canonical pathway; reduces lipid hydroperoxides to alcohols.	[46,47]
Layer 2	FSP1-CoQ10	Ubiquinol (CoQH_2_)	Plasma membrane	Independent of GSH; regenerates ubiquinol via NADPH.	[42]
	DHODH-CoQ10	Ubiquinol (CoQH_2_)	Mitochondrial inner membrane	Specific protection for mitochondrial membranes.	[43]
Layer 3	GCH1-BH4	Tetrahydrobiopterin (BH4)	Cytosol	Directly scavenges radicals and remodels fatty acids.	[44]
	DHCR7-7DHC	7-Dehydrocholesterol	Membrane lipids	7-DHC protects phospholipids from auto-oxidation.	[45]
Layer 4	MBOAT1/2	MUFA-Phospholipids	Plasma membrane	Remodels membrane lipidome; replaces PUFAs with MUFAs.	[48]

**Table 2:** Bidirectional effects of core ferroptosis regulatory factors in cancer.

Gene/Factor	Biological Role	Mechanism in Ferroptosis	Cancer Type(s)	References
*p53*	Tumor Suppressor	Transcriptional repression of SLC7A11; depletes GSH.	Breast, Lung, Colorectal	[65]
*CDKN2A*	Tumor Suppressor	Remodels lipid metabolism; increases PUFA-PL enrichment.	Glioblastoma (GBM)	[66]
*Merlin/YAP*	Tumor Suppressor	Activates ACSL4 transcription to increase peroxidizable substrates.	Mesothelioma	[67]
*iPLA2β*	Oncogenic/Evasion	Hydrolyzes peroxidized phospholipids; bypasses GPX4 defense.	Melanoma	[68]
*ACSL3*	Oncogenic/Evasion	Incorporates MUFAs (Oleic acid) into membranes; suppresses lipid peroxidation.	TNBC, Melanoma (Lymph)	[69]
*NFS1*	Oncogenic/Evasion	Maintains Fe-S cluster stability; prevents iron-starvation response in hyperoxia.	Lung Adenocarcinoma	[70]
*CD36*	Immuno-suppressive	Mediates fatty acid uptake in CD8+ T cells, triggering their own ferroptosis.	Various (TME)	[71]

## Data Availability

The original mass spectrometry data presented in the study are included in the article.
